# Notes and News

**Published:** 1896-01-11

**Authors:** 


### Notes and News.

(Collected and Communicated.)

The Earl of Stafford has accepted the presidency of the Throat Hospital, Golden Square.

The Hospital for Women, Soho Square, has received £2,000 from an anonymous donor, to permanently endow two beds in loving memory of his young wife.

The office of second assistant medical officer to the Chelsea Board of Guardians is open to women as well as men.

"Amicus" has just sent a cheque for £1,000 for the endowment of an "In Memoriam" bed in the London Homœopathic Hospital.

The in-patient department of St. John's Hospital for Skin Diseases, now situated in Leicester Square, is to be removed to Arlington House, in the Uxbridge Road, Shepherd's Bush. Accommodation for forty in-patients will be available.

H.R.H. The Duke of York has graciously consented to preside at a dinner in aid of the funds of the Victoria Hospital for Children, at a date to be fixed by His Royal Highness about the third week in February.

Charity is kind not only to its recipients but to those who use it as a means of attracting attention to their own good deeds. The advertising spirit is abroad, and we congratulate the Rev. Styleman Herring on the attractiveness of the invitation card issued last week to witness the departure of a hundred crippled and sick children for Sandgate. Why, however, people should be asked to come and stare at these unfortunates we do not know. We are glad they should have a holiday and a change, but we see no reason why their misfortunes should be made a show of for the sake of advertising either Mr. Herring or the London Samaritan Society or their convalescent home at Sandgate.

Cholera has not yet departed from St. Petersburg, but at Damietta it is fast diminishing. It has not spread elsewhere in Egypt except to a small degree in the province opposite Damietta.

The result of the last meeting of the Fellows of the Royal College of Surgeons makes matters look more hopeful for medical women. Mr. Clement Lucas's resolution, "That in the opinion of the Fellows of this college women should be admitted to the diplomas of the college," was carried by 47 to 10. It will be remembered that at a mixed meeting of members and Fellows in November last the same resolution was rejected by a majority of 10 out of 100 votes. It is held that the council of the college are not unwilling to accede to the application of the women, and therefore there is a prospect of its being accepted.

A meeting of the medical men of Bedford and district was held at the Infirmary on Thursday, January 2nd. Mr. Hughes Hemming, of Kimbolton, occupied the chair. It was decided to form a medical society in Bedford "for the promotion of professional intercourse, for the reading of papers, for the exhibition of cases, patients, pathological specimens, &c., and for the discussion of all questions affecting the welfare of the profession." Quarterly meetings will be held at the infirmary at half-past three p.m. Dr. G. P. Goldsmith was elected president; Mr. A. Chillingworth, treasurer; and Mr. W. Gifford Nash, secretary, for the ensuing year.

We rejoice to find that hospital committees and secretaries now afford evidence that they believe in the principle of appealing on the grounds that, if so much money is forthcoming, so much work will be done. An instance in point is the British Home for Incurables. The committee, owing to an enforcement of these principles, are able to commence the present year with a balance of £2,000 available for current expenses, after paying all the Christmas bills. In congratulating Mr. Salmond upon this excellent result, we feel confident it will be appreciated by the giving public, and that many will be induced by the facts here stated to extend their support to this excellent institution, because it is financed on business principles.

The *Pall Mall Gazette* is being wise in its generation, and in response to many inquiries as to the reason of the silence in its columns on the topic of its customary Christmas tree or other seasonable joys for the small ragamuffins of London, has let the public into deep plannings for future pleasures in store. Many good things have come to the poorer dwellers in the city this Christmas; trees and toys and food have been distributed far and wide, and it is doubtless as well that all the springs of benevolence should not be tapped at once. So our contemporary is biding its time, and will, with the return of long days and summer weather, call upon readers to contribute such a sum as shall suffice to set up "on some windy common or by the sea" a *Pall Mall* camp, to receive in hundreds for a brief space the little children of the streets. It is hoped thus to give some five thousand children a fortnight's fresh air and sunshine—a splendid idea, and one which will, when the time comes, no doubt receive the fullest and most generous support from the readers of the *Pall Mall Gazette*.

